# Assessing the condition of percutaneous coronary intervention services in Africa: challenges and prospects for advancement – a review

**DOI:** 10.1097/MS9.0000000000000924

**Published:** 2023-05-24

**Authors:** Nicholas Aderinto, Deji Olatunji

**Affiliations:** a Department of Medicine and Surgery, Ladoke Akintola University of Technology; b Department of Medicine and Surgery, University of Ilorin

**Keywords:** Africa, healthcare Infrastructure, percutaneous coronary intervention (PCI)

## Abstract

This study assesses the state of percutaneous coronary intervention (PCI) services in Africa, identifying the challenges and prospects for improvement. The study highlights limited infrastructure, resources, and the healthcare workforce as significant challenges in providing adequate PCI services to the population. However, opportunities for improvement are available through increased investment in healthcare infrastructure, healthcare professional training programs, and telemedicine. Collaboration among governments, healthcare providers, and international organizations is essential to address these challenges and improve access to high-quality PCI services for all Africans. Improving PCI facilities and utilization will not only benefit current and future patients with cardiovascular disease but will also advance healthcare as a whole in Africa.

## Introduction

Highlights

- Despite the high burden of coronary artery disease in Africa, the provision of percutaneous coronary intervention services is limited due to various challenges.
- These challenges include limited access to technology and skilled personnel, inadequate funding, and lack of proper infrastructure.
- The review highlights the need for partnerships with international organizations, government support, and increased investment in research and training programs to overcome these challenges.
- The paper concludes that by addressing these challenges, there is potential for significant advancements in percutaneous coronary intervention services in Africa, ultimately leading to improved health outcomes for patients with coronary artery disease.

Cardiovascular disease (CVD) is a major global health concern, with an estimated 17.9 million deaths attributed to CVD in 2019 alone^1^. Percutaneous coronary intervention (PCI), a minimally invasive procedure that unblocks narrowed or obstructed coronary arteries, is an important treatment option for patients with certain types of CVD, including acute coronary syndromes and stable angina^2^. In Africa, CVDs are the largest contributor to the total noncommunicable disease burden, accounting for 38.3% of noncommunicable disease deaths and 22.9 million disability-adjusted life years ^3^. However, the availability and quality of PCI services in Africa remain limited, with only a few countries on the continent having established PCI programs^4^. This lack of PCI services has significant implications for the management and outcomes of CVD patients in the region, as it can result in delayed or inadequate treatment, increased morbidity and mortality, and a lower quality of life.

Healthcare professionals in Africa face various challenges in providing adequate care to CVD patients, including a shortage of trained personnel, insufficient funding and resources, and inadequate infrastructure. For example, many PCI facilities in Africa have limited access to the latest technologies and equipment, such as drug-eluting stents and intravascular ultrasound, essential for delivering high-quality PCI services^5^. In addition, there is often a lack of coordination and integration between different levels of healthcare services, which can result in fragmented care and poor patient outcomes.

Against this backdrop, this narrative review aims to assess the current state of PCI services in Africa and to identify the challenges and prospects for their advancement. Specifically, the review will examine the current distribution and utilization of PCI facilities in Africa, the challenges healthcare professionals face in delivering effective PCI services, and the potential strategies and interventions for improving the availability and quality of PCI care in the region. By addressing these issues, this review aims to contribute to a better understanding of the state of PCI services in Africa and to inform the development of effective policies and programs for improving CVD outcomes in the region.

## Methodology

A comprehensive literature search was conducted to identify studies on the condition of PCI services in Africa. The search was performed using electronic databases such as PubMed, Medline, Scopus, and gray literature. The search terms included ‘PCI’, ‘coronary artery disease’, ‘Africa’, and ‘cardiovascular disease’. A total of 400 articles were initially identified through the literature search. The articles were screened based on their titles and abstracts, and duplicates were removed. A total of 50 articles were deemed relevant and retrieved for full-text review.

The full-text articles were assessed for inclusion based on the following criteria^1^: studies focused on the condition of PCI services in Africa^2^, studies published in peer-reviewed journals^3^, studies conducted between 2000 and 2023, and^4^ studies written in English. After applying these inclusion criteria, 36 articles were included in the final review. The articles excluded were not relevant to the topic of the review, were not published in peer-reviewed journals, were conducted outside the specified timeframe, or were not written in English.

### A review of existing literature

Based on current evidence, there is a significant difference in the availability and use of PCI facilities between developed and developing countries^6^. This results in limited access to proper care for patients with CVDs in developing countries, which can have negative effects on their health outcomes. A study found that the reperfusion rates for STEMI patients undergoing PCI varied significantly between countries, with South Africa reporting a rate of 59.7%, while Kenya reported only 13%^7^. However, these differences should be analyzed while taking into account variations in health coverage rates, technical support levels, and interventional cardiology teams. One major challenge is shortening the time between the onset of symptoms and admission to heart centers, with qualified emergency medical services(EMS) being scarce. To address this issue, the AFRICARDIO-2 meeting recommended specific goals to improve the management of STEMI in sub-Saharan Africa. These goals include increasing patient and primary care provider awareness, developing networks with cardiology referral facilities, and creating vascular closure devices^8^. Despite these efforts, the median distance from a PCI facility in Africa is 123.6 km, and the median driving time is 100 min^9^. This creates barriers to healthcare services, especially for low-income patients living in rural areas without medical insurance^10^. Additionally, there is widespread mistrust in EMS in Africa, with only a small percentage of high-acuity responses being serviced within the recommended time frame^11^. To improve reperfusion times, pre-hospital thrombolysis is recommended, and steps should be taken to recruit and retain paramedics in rural areas and to use helicopter emergency medical services for STEMIs^12^.

A study conducted over eight years in Uganda examined the experience of operating a single cardiac catheterization laboratory, providing tertiary cardiovascular services to 42 million children and adults^13^. The Uganda Heart Institute (UHI) conducted various diagnostic and interventional procedures, with 70% of the pediatric procedures being funded through philanthropic support. Both pediatric and adult procedures are financed through a co-pay system, where the government covers 50% of the procedure costs, and the patients and their families pay the remaining 50%. The study also highlighted challenges faced, such as technical expertise, training of interventional cardiologists, and a lack of staff. However, UHI was able to overcome these challenges by leveraging expertise from existing collaborations and the Ugandan government. A significant focus was on developing human resources, with nurses and technicians receiving training at centers in India, the US, and South Africa. However, the lack of backup equipment has resulted in gaps in service delivery, leading to a sharp decline in procedures performed in 2019. To decentralize specialized cardiology services to regional and district hospitals, a national health insurance scheme is required^14^. Currently, there is a lack of utilization of the available equipment.

A study conducted in Nigeria on stand-alone PCI at a catheterization laboratory demonstrated the potential for this procedure in Nigeria^15^. The study included 48 patients selected for PCI, with over 80% being urgent cases presenting with myocardial infarction and unstable angina. The complication rate was low, with only one patient experiencing a major adverse cardiac event, and there were no deaths resulting from PCI. These results suggest that there are experts ready and willing to perform these procedures if given the necessary resources and support.

In the cardiovascular journal of Africa, an editorial revealed that reperfusion can now be established using streptokinase (50%), target analytics (70%), primary percutaneous coronary intervention (PPCI), and stent placement (>90%) with varying degrees of effectiveness^16^. Studies have shown that there is an opportunity for incremental time-related myocardial salvage up to ~12 h after a patient with ST-elevation myocardial infarction (STEMI) presents^17,18^. The Abidjan Institute in Cote d’Ivoire was founded as a facility nearly 50 years ago to address this issue and is one of the rare but expanding numbers of such facilities in sub-Saharan Africa. Yao and colleagues provided observational data on their PPCI experience in STEMI patients hospitalized at their center over 10 years up to March 2019. More than 90% of PPCI patients presented within the first few hours of symptoms, and the hospital staff demonstrated excellent procedural success and low complication rates^7^. However, late presentations may be due to a cohort of patients who had already survived the riskiest early stages of their STEMI. The use of antiplatelet regimens and a more structured approach to left ventricular evaluations before and during reperfusion would be beneficial for the assessment of their STEMI program moving forward. It is also important to develop a strategy for late revascularization following myocardial infarction. For STEMI patients to benefit fully from a facility of this caliber, they must be sent there earlier and more frequently or receive thrombolysis in nearby clinics before being referred for PCI, also known as ‘facilitated’ PCI. Delayed referrals of STEMI patients are a problem everywhere but particularly severe in sub-Saharan Africa. The Stent-for-Life initiative sponsored by the European Society of Cardiology addresses this issue in Europe by helping to implement local STEMI treatment recommendations, identifying specific hurdles to adoption, and outlining steps to ensure that most STEMI patients receive the PPCI indication that can save their lives^19^.

The inadequate utilization of PCI in Africa has significant implications for the broader healthcare system and the management of CVD^20^. Firstly, Africa’s high burden of CVD results in a substantial increase in morbidity and mortality. Poor PCI utilization exacerbates this burden and lowers the quality of life for patients with CVD. Ischemic heart disease, in particular, contributes to mortality rates on the continent. By 2015, age-standardized mortality rates for IHD are expected to rise by 27 and 25% in African men and women, respectively, and by 70 and 74%, respectively, by 2030^21^. Secondly, the underutilization of PCI could eventually result in higher healthcare costs. Untreated CVD patients may develop complications that require more invasive and expensive treatments, such as coronary artery bypass grafting or heart transplants. Furthermore, the poor adoption of PCI may create significant disparities in healthcare access and outcomes between African countries and other regions internationally.

The role of race and ethnicity in CVD and the effectiveness of cardiac interventions, such as PCI, has been the subject of considerable research^22^. A growing body of evidence suggests that differences in the prevalence of certain risk factors for CVD exist across racial and ethnic groups^23^. For instance, hypertension has been found to be more prevalent among African patients compared to Asian and European patients in some studies, while differences in the prevalence of risk factors such as diabetes and smoking have also been observed across these groups^24^. Furthermore, research has suggested that there may be differences in the effectiveness of cardiac interventions among different racial and ethnic groups^25^. Specifically, studies have reported lower rates of PCI utilization and worse outcomes following PCI in African American patients compared to other racial and ethnic groups^26,27^. These disparities may be attributed to a range of factors, including access to care, socioeconomic status, and cultural beliefs and practices. Therefore, it is important to take a multifactorial approach to understanding and addressing cardiovascular health disparities in diverse populations. Nevertheless, the relationship between race, ethnicity, and cardiovascular health is complex and multifaceted. A variety of factors, including genetics, lifestyle behaviors, and environmental factors, also contribute to the development and management of CVD. As such, it is imperative to consider a holistic approach to address cardiovascular health disparities, taking into account not only race and ethnicity but also other relevant factors. This approach can help inform the design and implementation of interventions that can improve cardiovascular health outcomes in diverse populations.

### Challenges in delivering adequate percutaneous coronary intervention (PCI) care and treatment to patients with cardiovascular disease (CVD) in Africa

**Figure 1:**
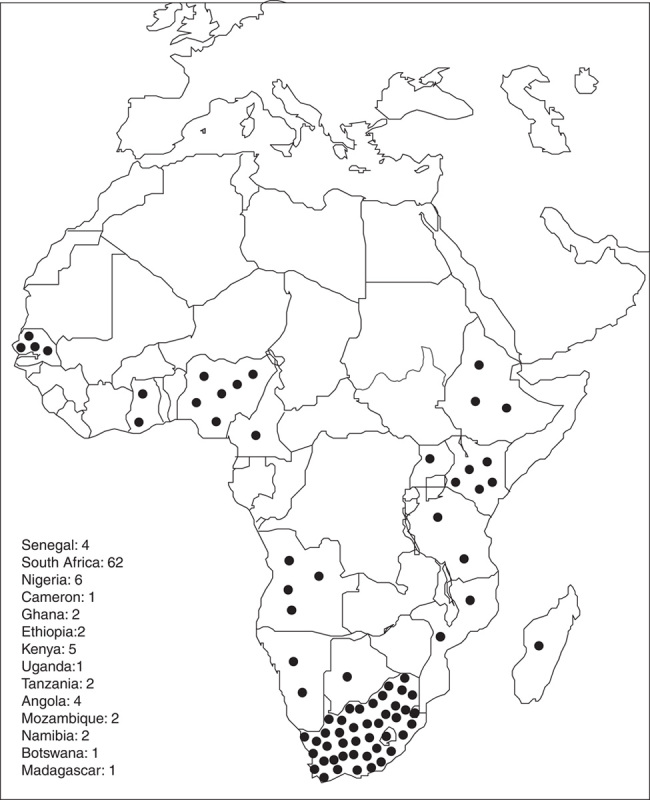
Cath lab distribution in Sub-Saharan Africa^29^.

Healthcare professionals in Africa face a significant challenge in providing adequate care and treatment for patients with coronary artery disease. One of the major contributing factors to this challenge is limited access to healthcare facilities that can provide effective PCI services^28^. In many African countries, these facilities are not widely available, making it difficult for patients, particularly those in rural areas, to receive the necessary care and treatment. Unfortunately, data on the number of available facilities is lacking, but it is evident that the number of PCI facilities in Africa is insufficient for the population^28^. For instance, South Africa, with a population of approximately 59 million people, has only 62 PCI facilities, making it challenging for most of the population to access them due to geographical and financial limitations^5^. See Figure 1.

In 2006, the region had only two functional cath labs, both located in Kenya, but this number increased to five by 2012, and there are currently only 12 cath labs spread across three countries in the region^6^. Sub-Saharan Africa has 32 cath labs, South Africa has 12, and South African provinces have 78, serving a population density of 22 million, 3.5 million, and 115 000 per lab, respectively^5^. This inadequacy is more apparent when compared to developed countries such as the United States, which maintains around 1700 catheterisation laboratories nationwide^28^. The limited access to PCI facilities in Africa has implications for patients’ quality of life, and it could result in higher healthcare costs as untreated CVD patients may develop complications that require more invasive and expensive treatments. Furthermore, it could create significant disparities in healthcare access and outcomes between African countries and other regions internationally. The European Society of Cardiology mapped the catheterisation laboratories within its member states and reported that Germany has 11.8 catheterisation laboratories per million people^29^. In comparison, Egypt recorded the lowest with 1.2 million catheterisation laboratories per million, the highest in Africa^6^.

The shortage of trained medical personnel in Africa, particularly in interventional cardiology, is a significant challenge that affects the quality of care provided to patients. Compared to the United States, where over 25 000 cardiologists serve a population of 328 million, Africa has only around 2000 cardiologists to serve a population of 1.2 billion^30^. This lack of personnel with the necessary skills and experience to perform complex procedures, such as PCI, is a major issue. In Nigeria, for example, there are only 500 general cardiologists to serve over 200 million people, and only a small fraction of them are trained to perform interventional procedures^31^. South Africa faces a similar challenge, with only 163 cardiologists serving a population of 55 million, and many provinces lacking any specialist cardiologists to provide services like PCI^32^.

Although professional training is available, it is limited to certain areas, and it can be prohibitively expensive for doctors to seek training abroad. Ozkan notes that the quality of professional training for cardiologists is generally high where it is available, ensuring that they are knowledgeable and skilled in their field^33^. However, in countries like Tanzania, Botswana, Swaziland, Lesotho, and Namibia, specialist cardiology training is almost nonexistent, leaving doctors with no choice but to seek foreign training, which can be prohibitively expensive^33^. The shortage of trained personnel is a significant barrier to expanding access to high-quality PCI services in Africa.

Financial challenges related to reimbursement for PCI procedures pose a significant obstacle for healthcare professionals in Africa. Due to inadequate investment in facilities and personnel, healthcare providers are often unable to provide high-quality care. This problem is compounded by a shortage of skilled personnel, which forces qualified professionals to relocate and offer their services elsewhere for better remuneration. Another challenge is the restricted availability of tools and materials necessary for PCI, including introducer needles, sheath introducers, guide catheters, radioopaque dye, guidewires, balloon catheters, stents, bare metal stents, and lithotripsy systems. These materials are frequently unavailable due to their high costs and difficulties with importation, limiting the number of patients who can receive treatment. In addition, financial restrictions prevent many African patients, particularly those without health insurance, from accessing PCI treatment. For instance, an uncomplicated chronic total occlusion PCI without physician fees costs ~$14 946, which is unaffordable for many individuals^34^. Moreover, the lack of a robust health funding structure, including health insurance, further compounds the problem by making it challenging for people to access EMS such as PCI in Africa.

## Recommendations

To improve the quality of care for patients with CVD in Africa, policymakers need to allocate more funds for PCI facilities and care, as well as for the prevention and treatment of the disease. See Table 1. The UHI in Kampala, which serves 40 million people and the four million residents of Kampala and its surrounding areas, recently received a $20 million loan from the OPEC Fund for International Development as part of a larger $73 million package^35^. This new center will provide specialized cardiac care services, enhancing the effectiveness and efficiency of cardiovascular care in the region.

**Table 1:** Problems and policy recommendations for improving PCI services in Africa

Problem	Policy recommendation
Limited access to technology	Increase investment in infrastructure and equipment and partner with international organizations to acquire advanced technology.
Limited availability of skilled personnel	Develop and implement training programs for healthcare professionals, and incentivise healthcare workers to practice in underserved areas.
Inadequate funding	Increase government funding for healthcare, and develop sustainable financing models.
Lack of proper infrastructure	Increase investment in healthcare infrastructure, including hospitals, clinics, and transportation systems.
Inequitable distribution of services	Develop policies and programs to improve access to care in underserved areas and promote health equity
Low patient awareness and uptake of services	Develop and implement health education campaigns to raise awareness about cardiovascular disease and the benefits of PCI services.
Limited availability of medications and equipment	Increase investment in pharmaceuticals and equipment and partner with international organizations to acquire necessary supplies.

**Figure 2:**
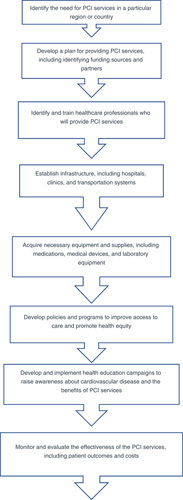
Organizing PCI services in Africa.

Governments and stakeholders should also invest in creating regional centers of excellence to serve as referral points for complex PCI cases. See Figure 2. These centers must be equipped with the latest technology and staffed by highly qualified medical teams. Research and innovation should be prioritized, including the development of new technology, refinement of treatment approaches, and clinical studies to assess different methods’ efficacy.

The Center for Cardiovascular Excellence at the Muhimbili University of Health and Allied Sciences in Tanzania is a notable example of an institution that has made significant progress in training specialists in CVD. The center achieved this by conducting training programs locally and internationally, in countries such as India, South Africa, Germany, the United Kingdom, and Ireland. A team of CVD experts from various domains was established to serve as the foundation for current and future CVD researchers at the center. The ultimate goal is to align these researchers with the center’s planned research program. Additionally, the University Medical Center Utrecht and the Center of Cardiovascular Excellence have signed a memorandum of understanding for advanced-level research training in tertiary CVD through joint PhD supervision at the University Medical Center Utrecht. Participants in this program receive comprehensive instruction in all aspects of scientific research, including research methodology, epidemiology, and scientific research writing, both in-person and online, in the Netherlands. All research following the proposed study agenda is conducted in Tanzania. This model can be replicated in other African countries to advance their capabilities in cardiovascular care and research.

In order to enhance access to PCI services, it is crucial to reach rural populations where the majority of people reside. One way to achieve this is by investing in mobile PCI units or expanding existing facilities to serve more patients. It is also essential to implement public awareness programs that educate people about the risk factors for CVD and the importance of seeking timely medical attention. Research has shown that such programs have a positive impact on CVDs, such as hypertension^36^. Similarly, increased awareness of myocardial ischemia events and the availability of PCI facilities can lead to similar effects. To improve the quality of care and inform policy-making, it is vital to have comprehensive data gathering and monitoring systems in place. This includes evaluating the prevalence of CVD, the utilization of PCI services, and treatment outcomes.

### Future directions for research

The advancement of PCI services in Africa requires a multifaceted approach that involves comprehensive research efforts aimed at understanding the clinical, economic, and social factors that shape the provision of care in this setting. To this end, several recommendations for future research have been proposed that can enhance the delivery and effectiveness of PCI services in Africa. It is important to conduct large-scale studies that examine the effectiveness of different PCI strategies in African populations, including the use of newer technologies and medications, which can generate critical evidence for guiding clinical practice and optimizing patient outcomes. These studies can also shed light on the unique clinical characteristics of African patients with coronary artery disease and inform the development of tailored treatment approaches that are effective in this population.

Furthermore, conducting cost-effectiveness analyses of PCI services in Africa can offer insights into the economic implications of delivering these interventions and inform resource allocation decisions and policy-making. This is particularly important in the context of limited healthcare resources in many African countries where cost-effective strategies for delivering quality care are essential for improving patient outcomes. In addition, developing and implementing quality improvement initiatives for PCI services in Africa can enhance the standard of care and reduce adverse outcomes. These initiatives can focus on various aspects of care delivery, including improving patient safety, enhancing communication between healthcare providers, and optimizing the overall quality of care.

Similarly, evaluating the long-term outcomes of PCI services in Africa can provide insights into the effectiveness and safety of these interventions over time. Such evaluations can include an assessment of morbidity, mortality, and quality of life measures, and can generate evidence for the long-term benefits of PCI. Also, conducting patient-centered research can provide valuable insights into the experiences and preferences of African patients with coronary artery disease regarding PCI services. Such research can inform the design and delivery of patient-centered care and help improve patient satisfaction with the care they receive.

### Clinical implications of review

The clinical implications of our study’s findings are noteworthy, emphasizing the need to improve access to high-quality PCI services in Africa, particularly for underserved populations, such as those residing in rural areas. Given the higher incidence of CVD in Africa, there is a pressing need to prioritize the development of PCI services in the region to reduce morbidity and mortality associated with coronary artery disease.

Efforts should be made to increase awareness and education about the benefits of PCI among healthcare providers and patients in Africa. This can be achieved through the development of evidence-based guidelines, educational initiatives, and public health campaigns that emphasize the importance of early diagnosis and timely intervention for coronary artery disease. Furthermore, our study highlights the potential of newer technologies and medications to improve the efficacy and safety of PCI procedures in African populations. Future research should focus on evaluating the effectiveness of these interventions in African populations to inform clinical practice and improve patient outcomes.

### Limitations of the Study

One limitation of this review is the language restriction, which excluded studies published in languages other than English. Therefore, some relevant studies may have been missed, potentially limiting the generalizability of the findings. Additionally, heterogeneity in study populations, including differences in sample sizes and patient characteristics across different African countries, may have impacted the applicability of the results. Despite these limitations, this review makes a valuable contribution to the literature by shedding light on the challenges that African countries face in providing PCI services. By highlighting these obstacles, the review offers important insights into the current state of PCI services in Africa and identifies promising strategies for improvement. These insights have significant implications for reducing the burden of CVD in the region and provide a useful framework for future efforts to improve PCI services in Africa.

## Conclusion

African nations encounter notable obstacles in providing sufficient PCI facilities to their citizens, mainly due to limited infrastructure, resources, and healthcare workforce. Nevertheless, there are prospects for progress through enhanced investment in healthcare infrastructure, healthcare professional training programs, and telemedicine. Governments, healthcare providers, and international organizations need to work together to tackle these challenges and enhance access to high-quality PCI services for all Africans. In addition to investing in healthcare infrastructure and workforce development, efforts should be made to increase public awareness and education about the benefits of PCI services in Africa. Public health campaigns that promote early diagnosis and timely intervention for coronary artery disease can be effective in reducing the incidence and severity of CVD in the region. Moreover, investing in PCI services can have a transformative effect on healthcare in Africa as a whole, improving the quality and accessibility of care for all patients. Improving PCI facilities and utilization in Africa will not only benefit current and future patients with CVD, but will also advance healthcare as a whole in the region. Through strategic investments in healthcare infrastructure, workforce development, and technology, African nations can improve access to high-quality PCI services and reduce the burden of CVD.

## Ethical approval

NA.

## Consent

NA.

## Sources of funding

None.

## Author contribution

N.A. and O.D.: conceptualization. All authors equally contributed in writing.

## Conflicts of interest

None.

## Research registration unique identifying number (UIN)

NA.

## Guarantor

NA.

## Data availability statement

NA.

## Provenance and peer review

Not commissioned, externally peer-reviewed.
